# Knowledge of preconception care among healthcare providers working in public health institutions in Hawassa, Ethiopia

**DOI:** 10.1371/journal.pone.0204415

**Published:** 2018-10-01

**Authors:** Andargachew Kassa, Sarie Petronella Human, Hirut Gemeda

**Affiliations:** 1 School of Nursing and Midwifery, College of Medicine and Health Sciences, Hawassa University, Hawassa, Ethiopia; 2 Departments of Health Sciences, College of Human Sciences, University of South Africa, (UNISA), Pretoria, South Africa; Centre Hospitalier Universitaire Vaudois, FRANCE

## Abstract

**Background:**

Preconception care (PCC) is an evidence-based health promotion intervention to prevent adverse pregnancy outcomes. Nevertheless, it is one of the missing elements within the continuum of maternal and child healthcare. Despite the WHO’s recommendation, most of the developing countries have not yet started implementing preconception care.

**Objective:**

To determine the knowledge level of healthcare providers about PCCand to identify predictors of effective knowledge of preconception care.

**Method:**

This is a cross-sectional study conducted among 634 healthcare providers (HCP) working in public health institutions of Hawassa. A pilot-tested and validated self-administered survey tool was used to collect data from individual healthcare providers who were selected randomly using a multistage sampling technique. The data entry and analysis were conducted using SPSS version 20 software. Frequency, proportions, means and standard deviations were used to describe the data. Bivariate and multivariate logistic regression models were implemented to determine the predictors of HCP’s PCC knowledge.

**Results:**

Only a few (31%) of the healthcare providers demonstrated a good level of knowledge on preconception care. The odds of having good PCC knowledge was high among HCPs working in hospitals (AOR = 1.8, 95% C.I. 1.3–2.6), HCPs using their smart phone to access clinical resources (AOR = 1.4, 95% C.I. 1.1–2.0), among those HCPs ever have read PCC guideline prepared by organization outside of Ethiopia (AOR = 1.9, 95% C.I. 1.4–2.7), among those who claimed practicing PCC (AOR = 3.4, 95% C.I. 2.0–5.9), and among those who earn salary of ≥ 146.0 $(AOR = 1.5, 95% C.I. 1.1–2.1).

**Conclusion:**

There is an unacceptably low level of knowledge about PCC among most of the healthcare providers in public health facilities in Ethiopia. The predictors identified in this study can be used to enhance the knowledge of healthcare providers about preconception care.

## Introduction

Providing preconception care (PCC) prior to conception by healthcare providers (HCPs) is a key intervention for healthier birth outcome[1]. Preconception care as defined by the World Health Organization (WHO) is“…*the provision of biomedical*, *behavioral and social health interventions to women and couples before conception occurs*. *It aims at improving their health status and reducing behavioural and individual and environmental factors that contribute to poor maternal and child health outcomes…”*[2]. The WHO strongly recommended the implementation of preconception care (PCC) five years ago. The organization also acknowledges the existence of PCC in some middle and lower income countries like Bangladesh, Philippines, Sri Lanka, and even in some African countries[2, 3]. The level of implementation of PCC in some of these African countries is varied[4–7]. The adoption of newer evidence-based innovations for change, like PCC, is still the challenge of the continent [8, 9]. Despite the availability of a number of evidence-based PCC clinical guidelines and recommendations, many of the low-income countries, including Ethiopia, have not yet introduced PCC in their national health systems[10].

Some studies from Europe, Canada, Iran and Egypt reported poor PCC knowledge and practice among HCPs [11–14]. Only one cross-sectional survey conducted in one of the African countries, Egypt, reported there is poor PCC knowledge and practice among the HCP’s. This study reported only 22% of HCPs had good knowledge[11]. Despite rigorous literature review and experience, the authors couldn’t find any evidence about the implementation of PCC in Ethiopia. The only published article that assessed women’s preconception knowledge in northern Ethiopia reported poor preconception health knowledge [15]. Nevertheless, the magnitude of adverse pregnancy outcome in Ethiopia is very high[2, 16]. For instance, the 2016 national demographic health survey reported maternal mortality ratio of 412/100,000 live birth, under child mortality rate of 67/1000 live birth, infant mortality rate of 45/1000 live birth, and Neonatal mortality rate of 29/1000 live birth[17].

The existing adverse pregnancy outcome preventive strategies in the country are highly reliant on delivery of antenatal care, institutional delivery, postnatal care, and child health services[16]. Integrating PCC in the routine practice of HCPs attending to women and men in their reproductive age is an effective strategy to initiate preconception care[10]. Healthcare providers are primarily responsible and at the forefront of incorporating up-to-date evidence-based clinical practices such as preconception care. Healthcare practitioners such as doctors, nurses, midwifes, and pharmacists need knowledge, a favourable attitude, and the necessary skills to provide PCC [2, 18, 19]. The level of implementation and integration of PCC in the health system in Ethiopia and the level of knowledge, attitude and skills of healthcare practitioners in this regard is still unclear. This study, therefore, aimed to assess the knowledge of healthcare providers about PCC and factors predicting good PCC knowledge[19].

## Method

The study was a cross-sectional quantitative study conducted from May to June 2017 among healthcare providers working in public health institutions (PHI) within the jurisdiction of Hawassa, 275km south of the capital (Addis Ababa) of Ethiopia. The public health institutions consist of nine healthcentres and two hospitals of which one was a secondary level public hospital and the other a tertiary level comprehensive specialized hospital. Under the public health centres are seventeen health posts where the health extension workers are working.

During the study period, healthcare workers consisted of 106 doctors, 826 nurses, 60 health officers, 95 midwifes, and 142 health extension workers who were employed in the institutions and formed the target population for the study. The health extension workers are primarily nurses, specifically trained to provide community health service in line with the country’s primary healthcare package. The maternal healthcare includes antenatal care, postnatal care and institutional delivery services. These services are provided in every health facility by all healthcare providers but mainly by midwives and gynaecologists. Preconception care is not a specified area of care in any of these facilities.

The authors of this study purposively selected Hawassa City Administration as the study area. Selection of the study area considered the goals of the study, feasibility issues, and the availability of all healthcare providers working at all levels of the referral system located at both rural and urban areas. The study sample of healthcare providers in public health institutions in Hawassa was randomly selected by using the employers register as a sampling frame. The sampled healthcare professionals were all taken proportional to their profession, their number, and the type of health facility where they are working. Healthcare workers who were employed for less than six months were excluded from the study.

Multistage sampling technique was applied to draw a total of 647 HCPs. The minimum sample size required for the study was determined by using a single population proportion formula. While computing the minimum sample size, the following parameters were considered: a 0.05 margin of error (α), a 95% Confidence Interval (CI), a 50% estimated proportion of healthcare providers’ knowledge about preconception care, 10% non-response rate and a design effect of 2. The design effect (DEEF) was calculated with the formula DEEF = 1+ *δ* (n-1). The “*δ” or the interclass correlation coefficient (ICC) was* calculated from the cluster data by using SPSS and it was found 0.169. Since the average size of clusters (n) was 11/2 = 5.5, the final DEEF was determinedas 1.79 ≈ 2. Given that the total number of HCPs working in PHIs of the study area was 1239, a population correction factor was considered. Due to the absence of a similar study or comparative study in the country we preferred taking a 50% proportion which is a proportion to yield adequate sample size.

Concerning sampling procedure, first, five PHIs out of the 11 PHIs found within the city administration were randomly selected. By using simple random sampling technique, 3 out of the 9 health centres were included in the study. Since the remaining two public hospitals were quite different in their level and type, both were selected. The study population was also stratified in terms of profession. In the second stage, HCPs were selected by the systematic random sampling method using employer’s employee registry document as a sampling frame. The study participants were all taken from each strata using probability proportional to size method. All HCPs were selected and consented to participate in the study without any coercion.

A data collection tool, namely *‘Andarg-Ethio PCC-KAP-Questionnaire for HCP’*, based on literature and evidence-based guidelines on PCC was developed and validated by the principal investigator to conduct this research project. The instrument was tested for content and face-validity by a panel of experts and was scored with a content validity index (CVI) of 92.4%. The reliability of the instrument was checked for its internal consistency with a Cronbach’s **α** test and demonstrated a score of 0.945[19]. The questionnaire which was originally prepared in English was translated to local language Amharic and then translated back to English. The survey was administered using the Amharic version.

The instrument was designed to assess socio-demographic characteristics of HCPs, their knowledge on PCC, their attitude towards PCC, their practice on PCC,issues on training of PCC,and in-service training opportunities on PCC. The HCPs’ knowledge of PCC was measured through 18 questions, each containing only one correct answer. A further 36 items measured various elements of PCC practice, including reproductive life planning,screening practices, access to resources to practice PCC. The attitude of healthcare workers on PCC was assessed by using 10 items each with five-point Likert scale responses.

The calculated single knowledge factor was then categorized into three ordinal categories. Respondents who scored less than the 50^th^ percentile or below the mean score were categorized as HCPs with ‘poor/low PCC knowledge’. Whereas, HCPs who scored ≥ 50^th^ percentile to 75^th^ percentile and those who scored > 75^th^ percentile were categorized as HCPs with ‘medium’ and ‘high’ PCC knowledge respectively. For analytical purpose, those HCPs who scored ‘high’ and ‘medium’ PCC knowledge were merged all together into another category called ‘*HCPs with good PCC knowledge’*.

The instrument was piloted on 65 (10% of the minimum sample) healthcare practitioners in a different town as Hawassa, after which minor revision to improve on the clarity of questions were done. The questionnaire was administered by two nurses, one health officer, one 2^nd^ year Master of public health student, and one pharmacist after being trained by the primary investigator. There were two field supervisors. The principal investigator was the main supervisor throughout the study.

The data collected was entered to the SPSS version 20 software by an experienced statistician. The analysis used descriptive statistics such as frequency, proportion, standard deviation, mean, mode and range to describe the variables of the study. In addition, the inferential statistics applied a binary and multiple logistic regression analytical models to determine the crude (COR) and adjusted odds ratios (AOR) respectively. The analysis was all made fixing CI at 95%. The variables with their P-Value of less than 0.20 were all considered in the second or multivariate logistic regression model. The second analytical step used a stepwise backward model to determine factors associated with ‘*HCP’s good PCC knowledge*’. The goodness of fit of the models was tested by using the Hosmer-Lemeshow test. Thus, the model which was found to be greater than the significance level (P-value = 0.05) was accepted.

The project proposal of the study was approved by IRBs of Hawassa University and the University of South Africa. Ethical principles such as confidentiality, beneficence, respect and human rights were maintained throughout the study.

## Results

### Socio-demographic characteristics of the sample population

The non-response rate (NRR)of the sample population was only 2%. Healthcare practitioners were mainly female389(61.4%). The majority (59.6%) of the participants were between 26–30 years of age and nurses by category (66.9%) (Table 1).

**Table 1:** Socio-demographic characteristics of healthcare providers working in public health institutions of Hawassa (n = 634), 2017, South Ethiopia.

Socio-demographic Characteristics	Total	
	Frequency (n)	Percent. (%)
**Sex**		
Female	389	61.4
Male	245	38.6
**Age**		
20–25 Years	178	28.1
26–30 Years	378	59.6
31–35 Years	55	8.7
> or = 36	23	3.6
**Marital status**		
Single	322	50.8
Married	300	47.3
Divorced	10	1.6
Widowed	2	.3
**Profession**		
Nurse	424	66.9
Health Extension Worker	62	9.8
Midwifery	57	9.0
Medical Doctor	51	8.0
Public Health Officer	40	6.3
**Education**		
Diploma	408	64.4
B.Sc.	187	29.5
M.Sc.	5	.8
GP/MD	28	4.4
Speciality/ MD	6	.9
**Year of experience**		
< 5 Years	381	60.1
> or = 5 years	253	39.9
**Monthly salary in birr**		
<146.0 $*	312	49.2
146.0–174.2$	179	28.2
>174.2$	143	22.6

*The exchange rate at the time of the study 1$ = 27.5 birr

### The level of the knowledge on preconception care

The level of the healthcare provider’s PCC knowledge score ranged from 0-16 (M = 9.44, SD = 2.4). Of the eighteen (18) items measuring knowledge, only 4 healthcare providers scored 0. There is no healthcare provider who scored 17 or 18 out of total questions. Despite their differences, nearly all study participants were aware of the benefits and the value of folic acid supplementation, adequate inter pregnancy spacing, prevention of mother to child transmission of HIV infection or PMTCT, family planning, nutrition, and stabilization of chronic disease before becoming pregnant. Almost half (43%) of the HCP had lower or poor knowledge about PCC and only (31%) of scored high on knowledge of PCC. The remaining 26% of the healthcare prodders are categorized as professionals with medium PCC knowledge.

### Predictors of good PCC knowledge

As shown in the table(Table 2), the likely hood of scoring good level of PCC knowledge was by about two times higher among healthcare providers working in public hospitalsthan those working in public health centres (AOR = 1.8, 95% C.I. 1.3–2.6). The likelihood of having good knowledge on PCC was also two-fold more when their monthly income was ≥146.0$ as compared to those who are paid less (AOR = 1.5, 95% C.I. 1.1–2.1). Healthcareproviders who are using a smartphone to access clinical resourcestested with higher levels of knowledge (AOR = 1.4, 95% C.I. 1.1–2.0).Those practitioners who have read guidelines or protocols on PCCprepared by other organization or from countries other than Ethiopia had odds of scoring good level of knowledge on PCC(AOR = 1.9, 95% C.I. 1.4–2.7). The factor with the higher odds to score among the listed factors was the actual practising of preconception care. Those care providers have claimed to practice PCChad odds of scoring a good level of PCCknowledge (AOR = 3.4, 95% C.I. 2.0–5.9).

**Table 2:** Bivariate & multivariate logistic regression analysis depicting predictors of good PCC knowledge among healthcare providers. 2017, Hawassa, South Ethiopia.

Factors	HCP’sknowledge on PCC		COR (95.0%C.I.)	AOR (95.0% C.I.)
	Good	Poor		
**Sex**				
Male	156	89	1.5(1.1–2.1) *	
Female	208	181	1^§^	
**Educational Level**				
B.Sc. & Above	29	10	2.3(1.1–4.7) *	
Diploma	335	260	1	
**Type of the PHI**				
Public Hospital	266	158	1.9(1.4–2.7) ***	1.8(1.3–2.6) **
Public Health centre	98	112	1	1
**Monthly income**				
≥ 146.0 $	200	122	1.5(1.1–2.0) *	1.5 (1.1–2.1) *
< 146.0 $	164	148		
**Use smart phone for sharing downloading & reading clinical resources**				
Yes	227	139	1.6(1.2–2.2) *	1.4 (1.1–2.0) *
No	137	131	1	1
**Ever read PCC guideline or protocol**				
Yes	203	112	1.8(1.3–2.5) ***	1.9 (1.4–2.7) ***
No	161	158	1	1
**Practicing PCC**				
Yes	78	19	3.6(2.1–6.1) ***	3.4 (2.0–5.9) ***
No	286	251	1	1
**Presence of Library in the PHI**				
Yes	216	106	2.3(1.6–3.1) ***	
No	148	164	1	
**Profession**				
Medical doctor	35	16	3.7(1.7–8.1)	
Nurse	248	176	2.4(1.4–4.1)	
Midwifery	33	24	2.3(1.1–4.9)	
Health Officers	25	15	2.8(1.2–6.4)	
Health Extension worker	23	39	1	

^§^ = Reference category

* = PV<0.05

** = PV<0.001

*** = PV<0.0001, COR = Crude Odds Ratio AOR = Adjusted Odds Ratio, CI = confidence interval

## Discussion

The knowledge of healthcare providers on PCC is the most important determinant of implementation of a healthcare intervention. Healthcare providers don’t usually practice what they don’t know [20]. The current study demonstrated poor PCC knowledge among the greater proportion (43%) and higher PCC knowledge among 31% of the healthcare providers working in public health institutions of Hawassa. This finding is nearly consistent with the findings in a survey conducted in Egypt that reported higher knowledge among only 22% of healthcare providers[11]. Another study conducted in Iran reported a moderate level of PCC among 30–67% of healthcare providers. According to the report, providers with poor knowledge of PCC were 11.7%[12]. Unlike Ethiopia, the implementation of PCC in Iran existed years before the reported research was conducted which resulted in health providers having being exposed to a longer period of access to information on PCC [21]. A study conducted in Ontario Canada reported poor PCC knowledge as one of the barriers of physicians to provide PCC[13].

In the current study, the level of PCC knowledge varies amongst the study participants with a range of knowledge score 0–16 (M = 9.44, SD = 2.4).The study also identified items equally and correctly responded by all healthcare providers who participated in the study. The similarities of awareness included the health benefits of folic acid supplementation, adequate inter-pregnancy spacing, prevention of mother to child transmission of HIV infection, family planning, nutrition, and stabilization of chronic disease before pregnancy. These commonalities may be linked to the inclusion of these preconception care-related topics that are included in curriculums and national guidelines. Whereas, the variation noted in the level of awareness may be due to the non-inclusion of standardized PCC training in pre-service courses in primary healthcare curricula [19].

Identifying factors or indicators in terms of the level of knowledge on PCC is vital input to programs targeting enhancement of provider’s knowledge. Literature on this study field did not report extensively on factors that impact on the knowledge levels of PCC amongst healthcare providers. The following indicators however can be predictive indicators of knowledge on preconception care: inclusion/non-inclusion of PCC in training curricula, the inclusion/ exclusion of PCC as part of in-service programmes; and the provision/absence of guidelines or protocols on PCC[2, 13, 22]. It was reported in one study that there is proportional variance in knowledge levels between healthcare providers with different professional backgrounds[2]. In the current study, however, professional background and educational level were not key predictors of their knowledge. This may be linked to the non-inclusion of PCC in training curricula in Ethiopia [19].

Policy document analysis conducted as part of this research project reveals the absence of PCC guidelines in the country[19]. Nevertheless, those who accessed PCC guidelines from sources outside of Ethiopia had a good knowledge level of PCC and this could be linked to the use of smartphones to access clinical resources. Using the traditional utilisation of library sources or presence of a library at the health institutions didn’t show any statistically significant association at the multivariate level. This may be linked to the absence of PCC guidelines in the facility or the underutilization of the library by healthcare provides. The value of m-health in the facilitation of clinical innovations and betterment of clinical performance or decision-making is well documented. Currently, the value of using mobile phones and internet as a mechanism for knowledge sharing among the Ethiopian healthcare providers is well recognized[16, 23]. This finding can be taken as an opportunity to enhance the knowledge of healthcare providers working in a resource-limited area and those working under a busy work schedule.

The HCP’s monthly income and the type of facility where the HCPs working were also among the factors affecting the HCP’s PCC knowledge. The analysis indicated that the likelihood of having good PCC knowledge was higher among HCPs who earn better salary and working in a hospital setting. The professional skill mix, the number of specialists and the types of clinical cases attended at public hospitals are quite different and by far better than public health centres[16]. This might have negatively affected the knowledge and skills of the HCPs working at health centres. Lower financial income can also affect the HCP’s potential to afford and access resources[23]. Those HCP’s paid lesser wages may not afford to have smart phones or to pay for internet. This might have negatively affected their knowledge sharing experiences. This finding implies the need to consider better mechanisms to enhance HCP’s access to standard e-resources and internet service. Mechanisms that increase the HCP’s access to online and offline PCC resources may increase the knowledge level of HCP’s.

Healthcare providers who claimed to have ever practiced PCC had higher odds of knowledgeabout PCC as compared to those who didn’t practice PCC before. There is sufficient evidence reported by implementation researchers that knowledge of a specific intervention is crucial to an evidence-based practice in that particular field, such as PCC [24, 25]. Providers offering PCC will, therefore, be more likely to implement PCC if they are provided with guidelines or protocols on PCC practice or have access to other sources of information in this field, such as electronic or published sources of information. Training of all healthcare providers in PCC practice and reinforcing provider-initiated PCC across all public health institutions will benefit both the healthcare provider as well as the healthcare consumers.

## Conclusion

There is poor knowledge among most of the healthcare providers. The proportion of healthcare providers who exhibited an acceptable level or higher PCC knowledge was 31%. About one fourth (26%) of the HCPs were found with medium or fair level of PCC knowledge. The remaining 43% had low or poor PCC knowledge. The odds of having good PCC knowledge was high among HCPs working in Hospitals, HCPs using their smart phone to access clinical resources, among those HCPs ever read PCC guideline prepared by an organization from outside of Ethiopia, among those who claimed to practice PCC, and among those who earn a salary of≥146.0$.

The value of this study is that it assessed the HCP’s knowledge of PCC and factors associated with their good PCC knowledge for the first time in Ethiopia. The study findings can serve as an input to enhance the practitioner’s knowledge on PCC and in so doing impact positively on pregnancy outcomes. It can also serve as baseline evidence to compare the results of future similar research findings and to assess the effect of a future corrective intervention on the HCP’s knowledge status. The identified factors associated with HCP’s good PCC knowledge can help program planners and decision makers who work towards changing the current providers PCC knowledge status.

As this is a cross-sectional survey, limitations linked to the study design are considered. The findings of this study show the status observed during the study period. Despite the proactive steps taken to reduce or limit their occurrences, the possibility of recall and social desirability bias are anticipated. The findings of this study may be applicable to HCPs working in other parts of the country; nevertheless, the generalization of the study findings beyond the target population needs prudence.

### Recommendations

Based on the findings of this study the following recommendations are forwarded.

- The Federal Ministry of Health, the Federal Ministry of Education and other concerned organizations and individual stakeholders are advised to improve the observed poor PCC knowledge of the HCP’s by providing continuous pre-service and in-service training.
- The Federal Ministry of Health and Regional health bureaus should consider to availing resources such as guidelines and other published information sources to increase the knowledge of HCPs working in the health centres by involving universities and other professional organisations.
- The authors recommend optimising the useof smartphone technology to download and sharing clinical practice like PCC and other guidelines to have up-to-date guidelines to practice or apply the evidence-based practice. Provision of access to technology as sources for information is highly recommended. This could take the form of an allowance to key stakeholders to use Internet or by providing Internet facilities in healthcare facilities
- Revising the nature and content of in-service training and providing mentorship in the implementation of PCC at facility level will promote a positive attitude and increased knowledge on preconception care.
- It will be valuable to consider conducting a similar survey across the country in an ongoing fashion. These can be helpful in monitoring and evaluation of future corrective interventions.
- The validated instrument used in this study can be used by other interested researchers.

## Supporting information

S1 File Amharic version questionaire. “Andarg-Ethio-PCC-KAP-Questionnaire for HCP’.
(DOCX)

S2 File English version questionaire. “Andarg-EthioPCC-KAP-Questionnaire for HCP’.
(DOCX)

## References

[1] ACP-ACOG. Guidelines for perinatal care (6th ed.). USA: American Academy of Paediatrics (ACP), American College of Obstetricians and Gynaecologists (ACOG); 2007.

[2] WHO. Meeting to develop a Global consensus on preconception care to reduce maternal and childhood mortality and morbidity. Geneva, Switzerland: World Health Organization; 2013.

[3] WHO. Preconception care: Maximizing the gains for maternal and child health: WHO: policy brief. Geneva, Switzerland: World Health Orgamization; 2013.

[4] Al-Darzi W, Al-Mudares F, Farah A, Ali A, Marzouk D. Knowledge of periconceptional folic acid use among pregnant women at Ain Shams University Hospital, Cairo, Egypt. Eastern Mediterranean health journal = La revue de sante de la Mediterranee orientale = al-Majallah al-sihhiyah li-sharq al-mutawassit. 2014;20(9):561–8. Epub 2014/10/25. . 25343469

[5] NASCOP. Guidelines on use of Antiretroviral Drugs for treating and preventing HIV infection: A rapid advice. Ministry of Health; National AIDS and STI Control Program 2014.

[6] Ajayi GO, Popoola AT, Dina T, Okorie N. Pre-pregnancy counseling in Lagos: a report on the first 1,000 cases. Clinical and experimental obstetrics & gynecology. 2013;40(3):359–60. Epub 2013/11/29. . 24283165

[7] MOH. Guidlines for maternity care in South Africa. 4th edition. Pretoria, South Africa: Department of Health Republic of South Africa 2015.

[8] Yuan CT, Nembhard IM, Stern AF, Brush JE Jr., Krumholz HM, Bradley EH. Blueprint for the dissemination of evidence-based practices in health care. Issue brief. 2010;86:1–16. . 20469542

[9] Bradley EH, Curry LA, Taylor LA, Pallas SW, Talbert-Slagle K, Yuan C, et al A model for scale up of family health innovations in low-income and middle-income settings: a mixed methods study. BMJ open. 2012;2(4). 10.1136/bmjopen-2012-000987 ; PubMed Central PMCID: PMC3432850. 22923624 PMC3432850

[10] Moos M-K, Dunlop AL, Jack BW, Nelson L, Coonrod DV, Long R, et al Healthier women, healthier reproductive outcomes: recommendations for the routine care of all women of reproductive age. American Journal of Obstetrics and Gynecology. 2008;199(6):S280–S9. 19081422 10.1016/j.ajog.2008.08.060

[11] Fadia M, Azza Refaat T, Emam E. Awareness of primary health care providers in Elminia Governorate about preconception care, Egipt. El-Minia Medical Bulletin. 2012;23(1):14.

[12] Bayram Ebrahimipour, Ebrahimi Froutani, Najafzadeh. Health care provider s’ knowledge, attitude and practice regarding pre-conception care. Journal of Research and Health. 2013;3(8).

[13] BSRC. Preconception Health Physician Practices in Ontario. Best Start: Ontario’s Maternal, Newborn and Early Child Development Resource Centre. Ontario, Canada: Best Start Resource Centre (BSRC), 2009.

[14] Braspenningx S, Haagdorens M, Blaumeiser B, Jacquemyn Y, Mortier G. Preconceptional care: a systematic review of the current situation and recommendations for the future. Facts, views & vision in ObGyn. 2013;5(1):13–25. ; PubMed Central PMCID: PMC3987351. 24753925 PMC3987351

[15] Ayalew Y, Mulat A, Dile M, Simegn A. Women's knowledge and associated factors in preconception care in adet, west gojjam, northwest Ethiopia: a community based cross sectional study. Reproductive health. 2017;14(1):15 Epub 2017/01/27. 10.1186/s12978-017-0279-4 ; PubMed Central PMCID: PMC5264481. 28122628 PMC5264481

[16] FMOH. The Federal Democratic Republic of Ethiopia Ministry of Health (FMOH) Health Sector Transformation Plan (HSTP) 2015/16–2019/20. Addis Ababa, Ethiopia: FMOH; 2015.

[17] ICF CSACEa. Ethiopia Demographic and Health Survey 2016. Addis Ababa, Ethiopia, and Rockville, Maryland, USA: CSA and ICF, 2016.

[18] Sanders LB. Preconception care: practice and policy implications for nurses. Policy, politics & nursing practice. 2009;10(2):129–33. 10.1177/1527154409338494 . 19783535

[19] Kassa A. Addressing the high adverse pregnancy outcomes through the incorporation of preconception care (PCC) in the health system of Ethiopia [PHD]. Pretoria, South Africa: University of South Africa (UNISA); 2017.

[20] McCluskey A. Occupational therapists report a low level of knowledge, skill and involvement in evidence-based practice. Australian Occupational Therapy Journal 2003;50(1):10 10.1046/j.1440-1630.2003.00303.x

[21] Sardasht FG, Shourab NJ, Jafarnejad F, Esmaily H. Application of Donabedian Quality-of-Care Framework to Assess the Outcomes of Preconception Care in Urban Health Centers, Mashhad, Iran in 2012. Journal of Midwifery Reprod Health. 2013;1(2):10.

[22] Kitamura K, Fetters MD, Ban N. Preconception care by family physicians and general practitioners in Japan. BMC family practice. 2005;6:31 Epub 2005/07/30. 10.1186/1471-2296-6-31 ; PubMed Central PMCID: PMC1184067. 16050958 PMC1184067

[23] Asemahagn MA. Knowledge and experience sharing practices among health professionals in hospitals under the Addis Ababa health bureau, Ethiopia. BMC Health Services Research 2014 14(431). 10.1186/1472-6963-14-431 PMC4263061 25253270

[24] Fleuren M, Wiefferink K, Paulussen T. Determinants of innovation within health care organizations: literature review and Delphi study. International journal for quality in health care: journal of the International Society for Quality in Health Care. 2004;16(2):107–23. Epub 2004/03/31. 10.1093/intqhc/mzh030 . 15051705

[25] Fleuren MA, Paulussen TG, Van Dommelen P, Van Buuren S. Towards a measurement instrument for determinants of innovations. International journal for quality in health care: journal of the International Society for Quality in Health Care. 2014;26(5):501–10. Epub 2014/06/22. 10.1093/intqhc/mzu060 ; PubMed Central PMCID: PMC4195468. 24951511 PMC4195468

