# Supplementary material for: Knowledge of preconception care among healthcare providers working in public health institutions in Hawassa, Ethiopia
Source: PLoS One. 2018 Oct 1;13(10):e0204415. doi: 10.1371/journal.pone.0204415 (PMC6166966; doi:10.1371/journal.pone.0204415)
Supplement: S1 File — “Andarg-Ethio-PCC-KAP-Questionnaire for HCP’. (DOCX) [file pone.0204415.s001.docx]

ይህንን ጥናት የምናከሂደው በሃዋሳ ዩኑቨርሲቲ ህክምናና ጤና ሳይንስ ኮሌጅ የምንሰራ ተመራማሪዎች ስንሆን የጥናቱም ርእስ **"በእርግዝና እና ወሊድ ወቅት በእናቱ እና በሚወለደው ጽንስ/ልጅ/ ላይ የሚከሰቱ የጤና ቀውሶች ለመከላከል የሚሰራውን የቅድመ ጽንሰት ጤና ክብካቤ አገልግሎት (ቅ/ጽ/ጤ/ክ/አ) ምንያህል እየሰሩ እነደሆነ ለማጤን የሚደረግ ጥናት"** ነው፡፡ የ ጽሁፍ ቃለመጠይቁ ከ5 እሰከ 10 ደቂቃ ሊፈጅ ይችላል፡፡ ጥያቄዎቹ ስለ ቅ/ጽ/ጤ/ክ አገልግሎት ያልዎትን ግንዛቤ የትም ዝግጅት እነዲሁም ምን ያህል እየሰሩ እነደሆነ የሚጠይቁ ናቸው፡፡ በዚህ ጥናት በመሳተፍዎ በቀጥታ የሚያገኙት ጥቅም ላይኖር ይችላል ሆኖም ጥናቱ በእናቶች በጽንስና በልጆች ጤንነት ላይ የሚደርሰውን ችግር በመቀነስ የእናቶችንና የልጆችን ሞት፣ ጭንገፋ፣ አካልጉዳተኝነት፣ እና ሌሎችንም ተያያዥ ችግሮች ለመቅረፍ እየተደረገ ያለውን ጥረት ለመቅረፍ ስለሚረዳ በተዘዋዋሪ እርሶንና ማህበረሰቡን የሚጠቅም ስራ ነደሚሆን ተስፋ እናደርጋለን፡፡ የምናገኘውን መረጃ በሚስጥር እንጠብቃለን፡፡ ስምዎ ተመዝግቦ አይያዝም፡፡ በጥናቱ ያለመሳተፍ ሙሉ መብትዎ የተጠበቀ ነው፡፡ ለመሳተፍ ፈቅደው አንዳንድ መረጀዎችን ላለመስጠት ከፈለጉ መረጀውን ያለመስጠት መብትዎ ይከበርልዎታል፡፡ ምናልባት መረጀውን ከሰጡ በሁዋላ ስለ ግል መረጃዎ ጉዳይ ወይም ስለጥናቱ ማወቅ ከፈለጉ የጥናቱን ዋና ተመራማሪ አንዳርጋቸው ካሣ (ረ/ፕ) በ +251-911-338895 በመደወል ወይም በሃዋሳ ዩኑቨርሲቲ ህክምናና ጤናሳይነስ ኮሌጅ ቢሮ ቁጥር 36 በስራቀን በአካል በመምጣት ማግኘትና መጠየቅ ይችላሉ፡፡

እባክዎ ፈቃደኝነትዎን በፊርማዎ ያረጋግጡል፡፡ ፊርማ_________

በጥናቱ ለመሳተፍ ስላሳዩት በጎ ፈቃደኝነት አስቀድመን ልባዊ ምስጋናችንን በአክብሮት እንገልፃለን፡፡

| **የቅድመ ጽንሰት/ቅድመ እርግዝና/ ጤና ክብካቤ (Preconception care (PCC) ምንነት/Definition**  **የቅድመ ጽንት** ወይም **ቅድመ እርግዝና** **ክብካቤ** በእንግሊዝኛው **Preconception care (PCC)** ተብሎ የሚጠራ ሲሆን **ከእርግዝና** ወይም **ከመፀነስ** **በፊት** የሚሰጥ የጤና ክብካቤ አገልግሎት ነው፡፡  ቅድመ ጽንት/ **ቅድመ እርግዝና/ ክብካቤ (PCC)** በተለይ ለማርገዝ እቅድ ያላቸውን ሰዎች ለይቶ በማውጣት ሰዎቹ አገልግሎቱን እነዲያገኙ *ያደርጋል፡፡* |
| --- |

**ለጤና ባለሞያዎች የተዘጋጀ የቅድመጽንሰስ/የ ቅድመ እርግዝና አገልግሎት(Preconception Care (PCC) መጠይቅ:**

**መመሪያ፡** እባክዎ ከተራቁጥር 1 እስከ መጨረሳ ድረስ የተዘረዘሩትን እያንዳንዱን ጥያቄዎች በጥንቃቄ በማንበብ መልስዎን ከተሰጡት አማራጭ መልሶች በመረጡት የመልስ ሳጥን ውስጥ የራይት ምልክት (*tick*) በማድረግ እነዲሁም ክፍት ቦታ /ዳሽ/ በተሠጣቸው ላይ መልስዎን በመጻፍ ይግለጹ

**ክፍል አንድ: ከማህበራዊ ከዲሞግራፊያዊና ከሞያዊ ስራ ጋር የተያያዙ ጥያቄዎች**

1. **ፆታ** 1. □ ወንድ 2. □ ሴት
2. **አድሜዎ ስንት ነው _______** ዓመት
3. **የጋብቻ ሁኔታ**
4. □ ያላገባ/ች 2. □ ያገባ/ች 3. □ የፈታ/ች 4. □ ባል/ሚስት የሞተበት/ባት 5. □ ያለጋብቻ አብሮ መኖር
5. **ሃይማኖት 1.** □ ኦርቶዶክስ 2. □ ፕሮቴስታንት 3. □ ሙስሊም 4 □ ካቶሊክ 5. □ ሌላ ካለ ይገለጽ ____ ____
6. **ሞያ/Profession:**
   1. □ ሜዲካል ዶክተር
   2. □ ነርስ
   3. □ ሚድዋይፍ
   4. □ ፐብሊክ ሄልዝ ኦፊሰር
   5. □ የጤና ኤክስቴንሽን ባለሙያ
   6. □ ሌላ ______________________
7. **የስራ ልምደ/Year of experience/** ___________ ዓመት
8. **በሞያዎ የደረሱበት ከፍተኛ የት/ም ደረጃ (የተጠናቀቀ)**
   1. □ ዲፕሎማ
   2. □ ቢኤስሲ (B.Sc.)
   3. □ ማስተርስ (M.Sc.)
   4. □ ጂፒ /GP MD
   5. □ ስፔሻሊስት /MD
   6. □ ፒኤችዲ/PhD
9. **የሚሰሩበት የጤና ተቋም አይነት**  1. □ ጤና ጣቢያ 2. □ ሆስፒታል 3. □ ጤና ኬላ
10. **በአሁኑ ወቅት ተመድበው የሚሰሩበት ዲፓርትምነት ወይም ኬዝ ቲም ምንድነው/ምንይባላል ?** ______________________
11. **የወርሃዊ ደሞዝዎ መጠን በብር** ___________ ብር/ በወር
12. **በቀን በአማካኝ ምን ያህል ተገልጋዮችን ያስተናግዳሉ?** ______ ሰው/በቀን

**ክፍል ሁለት: በ ቅድመ ጽንሰት ጤና ክብከቤ** (PCC) **ዙሪያ የተዘጋጁ የ ግንዛቤ ጥያቄዎች**

|  |  | **እውነት**  **True** | **ሃሰት**  **False** | **አላውቅ Don’t know** |
| --- | --- | --- | --- | --- |
|  | የ ቅድመ ጽንሰት ጤና ክብከቤ **የሚያቅፋቸው (Eligibles of PCC)** እድሜያቸው በፍሬያማ እድሜ (Reproductive age) ክልል ውስጥ ያሉትን ወንድና ሴቶችን ሁሉ ነው | 1 □ | 2 □ | 3 □ |
|  | የሚፈለገውን ውጤት ለማግኘት **ቅድመ ጽንሰት ጤና ክብከቤ መጀመር ያለበት** አራት ሳምንት ከመጸነስ በፊት ነው፡፡ | 1 □ | 2 □ | 3 □ |
|  | ከእርግዝና በፊት ያልታከመ Periodontal disease ላልተፈለጉ የእርግዝና ወጤቶች **Adverse Pregnancy Outcome** (**APO**) ያጋልጣል፡፡ | 1 □ | 2 □ | 3 □ |
|  | በቅርቡ ለማርገዝ ያቀደች የ **BMI** ልኬቷ **19.5** የሆነ ሴት ላልተፈለገ የእርግዝና ውጤት (**APO)** የተጋለጠች ናት፡ ስለሆነም ቢያንስ 20.0 እስክትሆን መጠበቅ ይኖርባታል፡፡ | 1 □ | 2 □ | 3 □ |
|  | በፍሬያማ እድሜ (Reproductive age) ክልል ውስጥ ያሉ ሴቶች ሁሉ በየቀኑ **0.4** **ሚ.ግ** **folic acid** ታብሌት መውሰድ አለባቸው | 1 □ | 2 □ | 3 □ |
|  | የ ቅድመ ጽንሰት ክብከቤ አገልግሎት የሚያካትታቸው መደበኛ **(routine) ላቦራቶሪ ምርመራዎች** Hgb, Hct, HIV, HBV, HIV, እና RPR ወይም VDRL ይጨምራል | 1 □ | 2 □ | 3 □ |
|  | በ ቅድመ ጽንሰት **genetic counseling** እና **screening** ስራ ወቅት ሃኪሙ (የጤና ባለሙያው) የ **sickle cell**  **hemoglobinopathies** ችግር ያለባት ሰው ካገኘ **carrier screening tests** ማድረግ ጠቃሚ መሆኑን ሊነግራት ይገባል | 1 □ | 2 □ | 3 □ |
|  | የ ቅድመ ጽንሰት ክብከቤ አገልግሎት የሚሰጥ ባለሞያ **የቅድመ እርግዝና የስኳር (DM) ና የደምግፊት (HPN)** ላለባቸው ሰዎች  አስቀድሞ **ህመሙን የመቆጣጠር (control)** የማድረግ አስፈላጊነትን ሊነግራቸው ይገባል | 1 □ | 2 □ | 3 □ |
|  | **Isotretinions**, **Valproic acid**, እና **Warfarin** **ቴራቶጀኒክ** ያላቸው መድሃኒቶች ሲሆኑ እነዚህን መድሃኒቶች የሚወስዱ ና በቅርቡ መጸነስ ለሚፈልጉ ሴቶች የመድሃኒት ለውጥ ሊደረግለቸው ይገባል | 1 □ | 2 □ | 3 □ |
|  | በ ቅርቡ ለመጸነስ የሚፈልጉ **የአስም በሽታ** ያለባቸው ሴቶች ከእርግዝናቸው አንድ ወር በፊትና ከጸነሱበት አስከ ሶስት ወር ጊዜ ድረስ ሳልቡታሞል /**Salbutamol**/ መውሰድ የለባቸውም | 1 □ | 2 □ | 3 □ |
|  | በ ቅድመ ጽንሰት ክብካቤ አገልግሎት ወቅት አስቀድሞ እነደ **depression**, **seizure disorder**, እና **phenylketonuria**  ያሉትነ ከመጸነስ በፊት መለየትና ማከም ያልተፈለገ የእርግዝና ውጤት(APO**)** እንዳይከሰት ይረዳል | 1 □ | 2 □ | 3 □ |
|  | የ ቅድመ ጽንሰት ወቅት **በቀን ሶሰቴ ራንደም ብለድ ሹገር ምርመራ** (**RBS test 3x/day**) ማድረግ የ **pre-gestational diabetes** ታማሚን የደም ስኳር መጠን በአስተማማኝ ሁኔታ መቆጣጠራችንን የሚያረጋግጥልን ተመራጭ (**Gold Standard**) የምርመራ አይነት ነው:: | 1 □ | 2 □ | 3 □ |
|  | ያልተፈለገ የእርግዝና ውጤትን(APO) ከመከላከል አኳያ ከ **ኢንፈሉዌንዛ ክትባት** በስተቀር, የሁማን ፓፒሎማ (**Human Papilloma** ) ፤ የሩቤላ (**Rubella**) እና የቫሪሴላ (**Varicella** ) ቫይረስ ክትባቶችን በእርግዝና ወቅት መሥጠት የተከለከለ (contraindicated) ነው:: | 1 □ | 2 □ | 3 □ |
|  | በቅርቡ የ መጸነስ ሃሳብ ላላቸው ሴቶች **ለ 30 ደቂቃ ደቂቃ የሚቆይ እስኪያልብ የሆነ መካከለኛ የአካል እንቅስቃሴ በሳምነት 5**  **ጊዜ** ማድረግ እንዳለባቸው በጤና ባለሙያው ሊነገራቸው ይገባል፡፡ | 1 □ | 2 □ | 3 □ |
|  | አልኮል እና ሲጋራ የሚጠቀሙ በቅርቡ የ መጸነስ እቅድ ያላቸው ሴቶች ከመጸነሳቸው በፊት የሚጠቀሙትን የ አልኮልና የሲጋራ መጠን እንዲቀንሱ ሊነገራቸው ይገባል | 1 □ | 2 □ | 3 □ |
|  | በ መጀመሪያዎቹ 12 የእርግዝና ሳምንታት እንጂ በቅድመ ጽንሰት ወቅት እንደ ጨረር፤ የአረም ማጥፊያ ፣ ሊድ፤ ሜርኩሪ ፣  ድመት ጋር ከሚደረግ ንክኪ እና ከመሳሰሉ አካባቢያዊ ጠንቆችና መጠበቅ ያልተፈለገ የእርግዝና ውጤት እንዳይከሰት  አይከለክልም | 1 □ | 2 □ | 3 □ |
|  | በቅርቡ በ **C/S** የወለደችን እናትበ ቀጣይ ለመውለድ ብትፈልግ ከ ቀድሞው C/S ቢያንስ በ **18 ወራት ዘግይታ** ማርገዝ  ይኖርባታል | 1 □ | 2 □ | 3 □ |
|  | የ **ኢንፈርቲሊቲ ምርመራ እና ህክምና** በቅድመ ጽንሰት ክብከቤ ውስጥ የሚካተት አይደለም | 1 □ | 2 □ | 3 □ |

**ክፍል ሦስት፡ የ ቅድመ ጽንሰት ክብካቤ አገልግሎት ትግበራን በተመለከተ**

1. **በእለት ተእለት ስራህ/ሽ ወቅት በስነተዋልዶ የእድሜ ክልል ያሉትን ታካሚዎችህን መቼ ልጅ የመውለድ ወይም ለመጸነስ እነደሚያስቡ እቅዳቸውን ትጠይቃለህ/ ትጠይቂያለሽ?**
   1. □ በፍጹም አድርጌ አላውቅም
   2. □ አልፎ አልፎ እጠይቃለሁ
   3. □ አንድ አንዴ እጠይቃለሁ
   4. □ ብዙግዜ እጠይቃለሁ
   5. □ ሁልጊዜ እጠይቃለሁ
2. **በእለት ተእለት ስራህ/ሽ ወቅት የታካሚዎችህን መቼ ልጅ ለመውለድ ወይም ለመጸነስ እነደሚያስቡ እቅዳቸውን የ ምትጠይቅ ከሆነ የትኞቹን ሰዎች ነው የምትጠይቀው ?** (ከ አንድ በላይ አማራጭ መስጠት ይቻላል ፡፡ ያልተጠቀሰም ካለ በመጻፍ መጨመር ቻላል)
   1. □ አዋቂ ወንዶችን ( ≥ 19 Years)
   2. □ እድሜያቸው ከ 12-18 ያሉ ታዳጊዎችን/ጎረምሶችን
   3. □ አዋቂ ሴቶችን 19-49 years
   4. □ ሌላ ካለ ይጠቀስ___________________

**3.1.** በእለት ተእለት ስራህ/ሽ **ለማርገዝ ለሚያስቡ/ለሚየቅዱ ሰዎች** ከ ጥያቄ 303.1 - 315 በተዘረዘሩት ነጥቦች ዙሪያ **ምክር ትሰጣለህ ?**

|  |  | **በፍጹም**  **Never** | **አልፎ አልፎ**  **Rarely** | **አንዳንድ ጊዜ**  **Sometimes** | **ብዙ ጊዜ**  **Often** | **ሁልጊዜ/**  **Always** |
| --- | --- | --- | --- | --- | --- | --- |
|  | ስለ ቤተሰብ እቅድ | 1 □ | 2 □ | 3 □ | 4 □ | 5 □ |
|  | አራርቆ ስለመውለድ | 1 □ | 2 □ | 3 □ | 4 □ | 5 □ |
|  | የ አካል እንቀስቃሴ | 1 □ | 2 □ | 3 □ | 4 □ | 5 □ |
|  | ስለ ሰውነት ክብደት | 1 □ | 2 □ | 3 □ | 4 □ | 5 □ |
|  | ስለ ምግብ (Nutrition) | 1 □ | 2 □ | 3 □ | 4 □ | 5 □ |
|  | ስለ አልኮል፣ ቶባኮና ሌሎች አነቃቂ እጾች (Psychoactive Substance use) | 1 □ | 2 □ | 3 □ | 4 □ | 5 □ |
|  | ስለ ፎሊክ አሲድ (Multivitamin containing Folic acid ) | 1 □ | 2 □ | 3 □ | 4 □ | 5 □ |
|  | ከመጸነስ በፊት ቀድመው የነበሩ ህመሞችን ወይም አዲስ የተከሰቱ ህመሞችን የማከም የማስተካከልና የመቆጣጠር አስፈላጊነት | 1 □ | 2 □ | 3 □ | 4 □ | 5 □ |
|  | ስለ ኤች አይቪና (STIs/HIV) ምርመራ ጠቀሜታ | 1 □ | 2 □ | 3 □ | 4 □ | 5 □ |
|  | በሃኪም ትእዛዝም ሆነ ያለሃኪም ትእዛእ የሚወሰዱ መድሃኒቶች ስለሚኖራቸው ጠንቅ | 1 □ | 2 □ | 3 □ | 4 □ | 5 □ |
|  | ስለ አካባቢያዊ ጠንቆች እና መራዦች (Enviromnental hazard & toxins) | 1 □ | 2 □ | 3 □ | 4 □ | 5 □ |
|  | ስለ መከላከያ ክትባቶች (Preventive Vaccines) | 1 □ | 2 □ | 3 □ | 4 □ | 5 □ |
|  | በቅድመ ጽንሰት ክብከቤ ወቅት የ ትዳር አጋርን ወይም የፍቅር ገደኛን/partner/ የማሳተፍ አስፈለጊነት | 1 □ | 2 □ | 3 □ | 4 □ | 5 □ |

**3.2**. በእለት ተእለት ስራህ/ሽ ወቅት **በቅርቡ ለማርገዝ ለሚየቅዱ ሰዎችን** ከ ጥያቄ 316 - 328 በተዘረዘሩት ነጥቦች ዙሪያ **የቅድመ ጽንሰት አጋላጮችን** /*preconception risk factors)* ለማግኘት **ምርመራ (*health assessment*** )ታደርገለህ/ታደርጊያለሽ ?

|  | የደንበኛውን…/ የደንበናውን… | **በፍጹም**  **Never** | **አልፎ አልፎ**  **Rarely** | **አንዳንድ ጊዜ**  **Sometimes** | **ብዙ ጊዜ**  **Often** | **ሁልጊዜ/**  **Always** |
| --- | --- | --- | --- | --- | --- | --- |
|  | ዲሞግራፊያዊ(Demographic) መረጃ/ information/ | 1 □ | 2 □ | 3 □ | 4 □ | 5 □ |
|  | የ ቀድሞ ጋይኒኮሎጂና ኦብስታትሪክ መረጃ /Past Obs & Gyn history/ | 1 □ | 2 □ | 3 □ | 4 □ | 5 □ |
|  | የቀድሞ ሜዲካል/ሰርጂካል/ ታሪክ (Past medical/surgical history) | 1 □ | 2 □ | 3 □ | 4 □ | 5 □ |
|  | ጀነቲክ ሂስትሪ (Genetic history/Family pedigree) | 1 □ | 2 □ | 3 □ | 4 □ | 5 □ |
|  | የጥርስ ህክምና ክትትል ታሪክ(History of dental care or checkup) | 1 □ | 2 □ | 3 □ | 4 □ | 5 □ |
|  | ማህበራዊ ታሪክና የባህሪ ዳሰሳ (Social history/Lifestyle behaviors) | 1 □ | 2 □ | 3 □ | 4 □ | 5 □ |
|  | ታካሚው በ አካባቢያዊ ጠንቆች እና መራዦች ስላለው ተጋላጭነት | 1 □ | 2 □ | 3 □ | 4 □ | 5 □ |
|  | የመድሃኒት ተጠቃሚነትና የአጠቃቀምን መረጃ | 1 □ | 2 □ | 3 □ | 4 □ | 5 □ |
|  | ስለ አመጋገብ ሁኔታ/Nutritional assessment (BMI) | 1 □ | 2 □ | 3 □ | 4 □ | 5 □ |
|  | የማህበራዊና ስነልቦናዊ (Psycho-social ) ምርመራ | 1 □ | 2 □ | 3 □ | 4 □ | 5 □ |
|  | የአካል ምርመራ (Physical examination) | 1 □ | 2 □ | 3 □ | 4 □ | 5 □ |
|  | የስራና አይነትና የስራ አይነት ሁኔታ መረጃ (Employment history) | 1 □ | 2 □ | 3 □ | 4 □ | 5 □ |
|  | የቀድሞ ክትባት ሁኔታና (Vaccination status) | 1 □ | 2 □ | 3 □ | 4 □ | 5 □ |

**3.3.** በእለት ተእለት ስራህ/ሽ ወቅት **በቅርቡ ለመጸነስ ለሚያስቡ ሰዎች** ከ ጥያቄ 329 - 338 የተዘረዘሩትን **ስራዎች በራስህም/ሽም በመስራት ይሁን አገልግሎቱን ሊያገኙ ወደሚችሉበት ክፍል ወይም ተቋምና ባለሙያ ጋር በመላክ አገክግሎቱን እዲያገኙ ታደርጋለህ/*ታደርጊያለሽ* ?**

|  |  | **በፍጹም**  **Never** | **አልፎ አልፎ**  **Rarely** | **አንዳንድ ጊዜ**  **Sometimes** | **ብዙ ጊዜ**  **Often** | **ሁልጊዜ**  **Always** |
| --- | --- | --- | --- | --- | --- | --- |
|  | ፎሊክ አሲድ ማዘዝ/ መስጠት (Folic acid prescription) | 1 □ | 2 □ | 3 □ | 4 □ | 5 □ |
|  | ሠብስታንሰ መጠቀም እነዲያቆሙ መርዳት/Substance use cessation/ ለምሳሌ.  እንደ አልኮል፣ ሲጋራ…ወዘተ… | 1 □ | 2 □ | 3 □ | 4 □ | 5 □ |
|  | ጽንስ የማይጎዳ መድሃኒት ማዘዝ ወይም በሌላ በማይጎዳ መለወጥ/substitution/ | 1 □ | 2 □ | 3 □ | 4 □ | 5 □ |
|  | መደበኛ (routine)የቅድመ ጽንሰት/ቅድመ እርግዝና ላቦራቶሪ ምርመራ ማሰራት | 1 □ | 2 □ | 3 □ | 4 □ | 5 □ |
|  | ጽንሱንና የእናቱን ጤንነት ሊጎዱ የሚችሉ ቆዩና(chronic) አዲስ የተከሰቱ ( acute) የጤና ችግሮችን መርምሮ መለየትና እና ማከም | 1 □ | 2 □ | 3 □ | 4 □ | 5 □ |
|  | በቅድመ እርግዝና ወቅት የነበሩ የቆዩ በሽታዎችን ከቀጣዩ እርግዝና በፊት መቆጣጠር | 1 □ | 2 □ | 3 □ | 4 □ | 5 □ |
|  | ክትባቶችን ከኃገሪቱ መመሪያ እና ከደንበኛው ሁኔታ አኳያ መከተብ | 1 □ | 2 □ | 3 □ | 4 □ | 5 □ |
|  | የእርግዝና ማረጋገጫ ምርመራ ማድረግ | 1 □ | 2 □ | 3 □ | 4 □ | 5 □ |
|  | ደንበኛውን አስፈላጊ ወደሆኑ ቦታዎች/ድርጅቶች ጋር ማገናኘት | 1 □ | 2 □ | 3 □ | 4 □ | 5 □ |
|  | የ ኤች አይቪ ምርመራ ማድረግ | 1 □ | 2 □ | 3 □ | 4 □ | 5 □ |

**ክፍል አራት፡ በተመረጡ የ ቅድመ ጽንሰት ክብከቤ ጉዳዮች ዙሪያ የ ባለሙያዎች የመስማማትና ያለመስማማት ሁኔታ ዳሰሳ**

**መመሪያ፡** እባክዎ እያንዳንዱን ከ ቁጥር 401 - 410 ያሉትን ጥያቄዎችን ካነበቡ በሁዋላ በቀረበው ሃሰብ ላይ ምን ያህል እነደሚስማሙና እነደማይስማሙ ከ ጥያቄ ቁጥር 1-5 በተሰጡት አማራጮች ሳጥኖች ውስጥ ጭረት በማድረግ ይግለፁ፡፡ ( የምላሾቹ አይነቶች 1 □ **በጣም አልስማማም፣ 2** □ **አልስማማም ፣ 3** □ **አልወሰንኩም ፣ 4** □ **እስማማለሁ ፣ እና 5** □ **በጣም እስማማለሁ ናቸው)**

|  |  | **በጣም አልስማማም**  **Strongly**  **Disagree** | **አልስማማም**  **Disagree** | **አልወሰንኩም/**  **Undecided** | **እስማማለሁ/**  **Agree** | **በጣም**  **እስማማለሁ/**  **Strongly**  **Agree** |
| --- | --- | --- | --- | --- | --- | --- |
|  | የ ቅድመ ጽንሰት ክብከቤ አለመስጠት/ማጓደል/ ጽንሱን፣ ልጁን፣እናቱን በቀላሉ ሊመለስ ለማይችል የጤና ጠንቅ ወም ጥፋት ሊያጋልጥ ይችላል | 1 □ | 2 □ | 3 □ | 4 □ | 5 □ |
|  | የ ቅድመ ጽንሰት ጤና ክብከቤ የሴቶችን ጤና የተሻለ ለማድረግ ይረዳል | 1 □ | 2 □ | 3 □ | 4 □ | 5 □ |
|  | እንደ ኢትዮጵያ ባሉ ድሃ ሃገራት ለሚኖሩ ሰዎች የ ቅድመ ጽንሰት ክብከቤ  ለመስጠት ማሰብ ከቅንጦት ይቆጠራል | 1 □ | 2 □ | 3 □ | 4 □ | 5 □ |
|  | ሆስፒታል የ ቅድመ ጽንሰት ክብከቤ(PCC) ለመስጠት ተመራጭ ቦታ አይደለም | 1 □ | 2 □ | 3 □ | 4 □ | 5 □ |
|  | እንደ ኢትዮያ ባሉ ሃገራት የ ቅድመ ጽንሰት ክብከቤ አትኩሮት ጤናማ ለሆኑ  ሰዎች ሳይሆን እንደ HIV እና HBV በመሳሳሉ በሽታ ለተያዙት ብቻ መሆን  አለበት | 1 □ | 2 □ | 3 □ | 4 □ | 5 □ |
|  | የ ቅድመ ጽንሰት ጤና ክብከቤ መስጠት ሞያዊ ሃለፊነቴ አይደለም | 1 □ | 2 □ | 3 □ | 4 □ | 5 □ |
|  | ካለብኝ ተደራራቢ የስራ ጫናና በየቀኑ ከማስተናግዳቸው በርከት ያሉ ፈጣን  የህክምና እርዳታ ከሚፈልጉ የህሙማን ቁጥር አንጻር የ ቅድመ ጽንሰት ጤና  ክብከቤ ቅድሚያ የምሰጠው የስራ ድርሻዬ አይደለም፡፡ | 1 □ | 2 □ | 3 □ | 4 □ | 5 □ |
|  | የ ቅድመ ጽንሰት ጤና ክብከቤ የሚያስፈልጋቸው ጤናማዎቹ ወይም ጤናማ  የሚመስሉ ሰዎች ብቻ ሳይሆኑ ፈጣን የህክምና እርዳታ በሚያስፈልገው ህመም ለታመሙ ሰዎችም ጭምር ነው፡፡. | 1 □ | 2 □ | 3 □ | 4 □ | 5 □ |
|  | ሁሉም የጤና ባለሞያዎች የ ቅድመ ጽንሰት ጤና ክብከቤን ለሚያስፈልጋቸው  ደንበኞቻቸው በሙሉ ከሚሰሩት ስራ ጋር በቀላሉ አቀናጅተው ሊሰጡ  ይችላሉ፡፡ | 1 □ | 2 □ | 3 □ | 4 □ | 5 □ |
|  | የ ቅድመ ጽንሰት ጤና የስነተዋልዶ እና የ ሰብአዊ መብት ጋር የማይነጣጠሉ  ክፍሎች ሲሆኑ ስለዚህም ህሙማን ስለ ጉዳዩ ባለማወቅና አገልግሎቱን በማጣት ለሚደርስባቸው ችግር ተጠያቂ የሚሆኑት የጤና ባለሞያዎች ናቸው | 1 □ | 2 □ | 3 □ | 4 □ | 5 □ |

**ክፍል አምስት: ሌሎች ከ ቅድመ ጽንሰት ጤና ክብከቤ ጋር የተያያዙ ጉዳዬች**

|  |  | **አዎ በ ኮሌጅ ወይም ዩኑቨርሲቲ ቆይታዬ ወቅት** | **አዎ በስራ ላይ እያለሁ** | **አልተማርኩም/**  **አልሰለጠንኩም** | **አላስታውስም** |
| --- | --- | --- | --- | --- | --- |
|  | በእለት ተእለት ስራ ወቅት የምታክማቸውን ታካሚዎችን/ ጥንዶችን/ ልጆች የመውለድ እቅድ መጠየቅና ግልጽና አጭር ምክር ስለመስጠት (Reproductive life plan screening & brief counseling) | 1 □ | 2 □ | 3 □ | 4 □ |
|  | የማህበረሰቡን በ ቅድመ ጽንሰት ጤና እና ክብካቤ ዙሪያ ያለውን ግንዛቤ የማሳደግ ጠቀሜታ | 1 □ | 2 □ | 3 □ | 4 □ |
|  | ለማርገዝ ያቀዱ ሰዎች የፅንሱን እና የእናቱን ጤና ሊጎዳ የሚችል ጠንቅ እንዳላቸው የመለየት ምርመራ (how to conduct preconception risk assessment ) | 1 □ | 2 □ | 3 □ | 4 □ |
|  | ስለ የቅድመ ጽንሰት ጤና ት/ም እና ምክር አገልግሎት አሰጣጥ (how to provide preconception  educational & counseling) | 1 □ | 2 □ | 3 □ | 4 □ |
|  | በምርመራ ለተለዩ የቅድመ ጽንሰት ችግሮች ህክምና እና የማስተካከያ መንገዶቻቸው (how to manage identified preconception risk factors) | 1 □ | 2 □ | 3 □ | 4 □ |
|  | በእርግዝናዎች መካከል ስለሚደረግ የቅድመ ጽንሰት ክብካቤ አገልግሎት አይነቶችና ስለ አገልግሎቱ አሰጣጥ  (the elements of interconception care needed to prevent APO) | 1 □ | 2 □ | 3 □ | 4 □ |
|  | ስለ ኤችአይቪ ኤድስ ምርመራ ወይም ህክምና (ለምሳሌ፡ PMTCT, PIHCT፣ VCT, or ART) | 1 □ | 2 □ | 3 □ | 4 □ |
|  | የቆየ ሕመም ላለባቸው ደንበኞች የ ቅድመ ጽንሰት የህክምና እና ክትትል/followup አገልግሎት (PCC  considerations for clients with other chronic diseases ) | 1 □ | 2 □ | 3 □ | 4 □ |
|  | የአልኮል ወይም፣ የቶባኮ ሱስ ያለባቸውን ሰዎች እነዲያቆሙ የሚያስችል ህክምና አሰጣጥ (how to provide alcohol or tobacco cessation service ) | 1 □ | 2 □ | 3 □ | 4 □ |

**ከ ጥያቄ ቁጥር 501 - 509 በተዘረዘሩት ርእሶች ዙሪያ ጽ/ምህርት ወይም ስልጠና ወስደሃል/ሻል?**

**ክፍል ስድስት፡ የቅድመ ጽንሰስ አገልግትን ለመስጠት የሚያግዙ ግብዐቶችን እና አገልግሎት አሰጣጥን በተመለከተ**

|  |  | **አዎ/ Yes** | **አይ/ No** |  |
| --- | --- | --- | --- | --- |
|  | የ ኢንተርኔት አገልግሎት ትታገኛለህ / ታገኚአለሽ? | □ 1 | □ 2 | "አይ " ከሆነ ወደ ጥያቄ ቁ 603 |
|  | የኢንተርኔት አገልግሎት የምታገኝ/ኚ ከሆነ ከየት ታገኛለህ / ታገኚአለሽ?  □ 1. ከ መሰሪያ ቤት / ላይብራሪ/  **(ከ አንድ በላይ አማራጭ መስጠት/ማክበብ ይቻላል፡፡)** □ 2 ከ ኢንተርኔት ካፌ በክፍያ  □ 3 ከ ሆቴሎች ዋይፋይ ሰርቪስ  □ 4 በግል የሞባይል/CDMA/ የኢንተርኔት |  |  |  |
|  | በስማርት ፎን(Smart Phone/SP/) ለስራ የሚያግዙ መረጃዎችን ትለዋወጣለህ/ትለዋወጪያለሽ? | □ 1 | □ 2 | □ 3 ስማረት ፎን የለኝም |
|  | መስሪያቤታችሁ ላይብራሪ አለው ? | □ 1 | □ 2 |  |
|  | መስሪያቤታችሁ የቅድመጽንሰት አገልግሎት አሰጣጥን የሚመራ የፖሊሲ፣ የፕሮሲጀር ዶክመንት፣ ወይም  ጋይድላይን አለው ? | □ 1 | □ 2 | □ 3 አላውቅም |
|  | ከዚህ ቀደም ከየትኛውም ምንጭ የቅድመጽንሰት አገልግሎት አሰጣጥ ጋድላይን ወይም ፕሮቶኮል (PCC  gridline or protocol) አይተህ ታውቃለህ ? | □ 1 | □ 2 |  |
|  | በ ኢትዮፕያ ጤና ጥበቃ ሚኒስትር (FMOH) የተዘጋጀ የቅድመጽንሰት አገልግሎት አሰጣጥ ጋድላይን  ወይም ፕሮቶኮል (PCC gridline or protocol) አይተህ ታውቃለህ ? | □ 1 | □ 2 | □ 3 አላውቅም |
|  | በመስሪያቤትህ የቅድመ ጽንሰት አገልግሎት የሚሰጥ ባለሞያ አይተህ ታውቃለህ ? | □ 1 | □ 2 |  |
|  | የቅድመ ጽንሰት አገልግሎትን እለት ከእለት በምትሰጠው የህሙማን/የደንበኞች/ አገልግሎትህ/ሽ ጋር  አቀዳጅተህ/ሽ ለመስጠት ፈቃደኛ ነህ/ሽ ? | □ 1 | □ 2 | □ 3 አልወሰንኩም |
|  | የ ቅድመ ጽንሰት አገልግሎት ስልጠና (PCC training )እነዲሰጥህ ትፈልጋለህ ? | □ 1 | □ 2 |  |

1. የ ቅድመጽንሰት ጤና አገልግሎት ሊሰጡ የሚገባቸው እነማን ናቸው? **(ከ አንድ በላይ አማራጭ መስጠት/ማክበብ ይቻላል፡፡)** ያልተጠቀሰም ካለ በመጻፍ መጨመር ይቻላል)
   1. □ ሁሉም ስፔሻሊስት ሃኪሞች
   2. □ ሁሉም አጠቃላይ ሃኪሞች
   3. □ ሁሉም ነርሶች
   4. □ ሁሉም ሚድዋይፎች
   5. □ ሁሉም የጤና መኮንኖች
   6. □ ሁሉም የ ከተማ ጤና ኤክስቴንሽን ባለሞያዎች
   7. □ ሁሉም የ የገጠር ጤና ኤክስቴንሽን ባለሞያዎች
   8. □ ሌላ ካለ ይጠቀስ ___________________
2. የ ቅድመ ጽንሰት ጤና ክብካቤ አገልግሎት በየትኛው የጤና ድርጅት ሊሰጥ ይገባዋል ብለህ ሃሳብህን/ሽን ትሰጣለህ/ትሰጪያለሽ? (ከ አንድ በላይ አማራጭ መስጠት ይቻላል ፡፡ ያልተጠቀሰም ካለ በመጻፍ መጨመር ይቻላል)
   1. □ ጤና ጣቢያ
   2. □ ሖስፒታል
   3. □ ሌላ ካለ ይጠቀስ ___________________
3. የ ቅድመጽንሰት ጤና አገልግሎት **ሰጥተህ/ሽ የምታውቅ/ቂ** **ከሆነ** በባለፈው ሶስት ወራት ውሰጥ በ ግምት ለስንት ሰዎች ሰጥተሃል/ሻል? ለ ________ ሰዎች

**በጥናቱ ፈቃደኛ ሆነው ስለተሳተፉ ልባዊ ምሰጋናዬን እገልጻለሁ!!!**

**____________________________________________________________**

| **General Information (To be filled by Research Assistant and Supervisors)** | | |
| --- | --- | --- |
| Health Facility Name |  | Are all pages checked for availability and completeness? |
| Date the questionnaire was completed | /_________/ 2017 | Remark by Datatec collector |
| Data collector name  Signature | ____________ |  |
| HP’s Department | ______________________________ |  |
| Codes/PHI – UCSC(Eg. 00/00/ - 000) | __________/___________/ - ______________ | Remark by Supervisor |
| Name of the Supervisor  Signature | ____________ |  |
| Date checked by supervisor | _____/_______/ 2017 |  |
