# Supplementary material for: Knowledge of preconception care among healthcare providers working in public health institutions in Hawassa, Ethiopia
Source: PLoS One. 2018 Oct 1;13(10):e0204415. doi: 10.1371/journal.pone.0204415 (PMC6166966; doi:10.1371/journal.pone.0204415)
Supplement: S2 File — “Andarg-EthioPCC-KAP-Questionnaire for HCP’. (DOCX) [file pone.0204415.s002.docx]

Thank you for dedicating approximately 5-10 minutes to the completion of this questionnaire. Your honest and accurate responses will contribute towards addressing the high maternal and infant mortality rate in our country. Based on your valued inputs, I will use the information to develop guidelines for better integration of ***preconception care (PCC)*** in our existing maternal and child health services. You can be confident that your input will remain confidential and will not expose you in any way.

| Definition of preconception care (PCC)  Preconception care (PCC): What is PCC?  ***Preconception care*** is the provision of biomedical, behavioral and social health interventions necessary to improve pregnancy outcomes and the overall health of women and her partner.  ***Preconception care (PCC)*** is among the parts of the continuum of maternal & reproductive health care services given ***before conceptions*** and ***between successive pregnancies*** (Inter-conception Care). |
| --- |

**Preconception Care (PCC) questionnaire: Health workers**

**Instruction:** *please read the following questions listed from Qn# 101 to 631 and tick in the box of the best option you choose and write your answer on the space provided to some of the questions.*

**PART ONE: Socio-demographic, professional, and work related questions**

1. **Gender** 1.1. □ Male 1.2. □ Female
2. **Your age in years _______** year
3. **Marital status**
4. □ Single 2. □ Married 3. □ Divorced 4. □ Widowed 5. □ Living together
5. **Religion 1.** □ Orthodox 2. □ Protestant 3. □ Muslim 4 □ Catholic 5. □ Other**_____**
6. **Profession:**
   1. □ Medical Doctor
   2. □ Nurse
   3. □ Midwife
   4. □ Public Health Officer
   5. □ Urban Health Extension Worker
   6. □ Other ______________________
7. **Year of experience/** ___________ y**ear**
8. **Maximum educational level attended with your health profession**
   1. □ Diploma
   2. □ B Sc
   3. □ M.Sc.
   4. □ GP MD
   5. □ MD/ Specialty
   6. □ PhD
9. **Practice setting**  1. □ Health center 2. □ Hospital 3. □ Health Post
10. **In which department you are currently working?** ______________________
11. **Monthly salary paid** in **Ethiopian birr** ________ Birr/month
12. **How many patients you manage per day at average?** ______ patients/day

**PART TWO: Preconception Care (PCC) Knowledge related questions.**

|  |  | **True** | **False** | **Don’t know** |
| --- | --- | --- | --- | --- |
|  | The eligible clients for preconception care (PCC) include all adolescents and  reproductive aged individuals | 1 □ | 2 □ | 3 □ |
|  | To be effective PCC should start four weeks before conception | 1 □ | 2 □ | 3 □ |
|  | Periodontal disease is a risk factor for adverse pregnancy outcomes (APO) | 1 □ | 2 □ | 3 □ |
|  | Women with BMI ≤ 18.4 planning pregnancy are at risk of developing APO | 1 □ | 2 □ | 3 □ |
|  | All women of reproductive age should take 0.4 mg (400 mcg) of folic acid daily. | 1 □ | 2 □ | 3 □ |
|  | The recommended routine pre-conceptual laboratory tests include Hgb, Hct, HIV, HBV,  HIV, and RPR or VDRL tests | 1 □ | 2 □ | 3 □ |
|  | Preconception genetic counseling and screening include recommending carrier screening  tests for client with sickle cell hemoglobinopathies | 1 □ | 2 □ | 3 □ |
|  | A clinician providing PCC for clients with diabetes mellitus and chronic hypertension  should recommend genetic screening testing | 1 □ | 2 □ | 3 □ |
|  | Isotretinions, Valproic acid, and Warfarin are medications poses teratogenic effects requiring preconception modification | 1 □ | 2 □ | 3 □ |
|  | Women with asthma planning pregnancy should avoid taking Salbutamol one month  before and after conception | 1 □ | 2 □ | 3 □ |
|  | Early identification and treatment of diseases like depression, seizure disorder, and  phenylketonuria during the preconception period reduce the occurrence of APO | 1 □ | 2 □ | 3 □ |
|  | The recommended test that guarantee good periconceptional blood sugar control for a  woman with pre-gestational diabetes is random blood sugar (RBS) test | 1 □ | 2 □ | 3 □ |
|  | Except Influenza vaccine, vaccines such as Human Papilloma virus, Rubella, and  Varicella are all vaccines contraindicated during pregnancy | 1 □ | 2 □ | 3 □ |
|  | Recommending regular exercise is an important PCC counseling point. Thus, a women  planning pregnancy should aim 30 minutes of moderate exercise 5 days a week. | 1 □ | 2 □ | 3 □ |
|  | Women planning pregnancy should be advised to delay pregnancy until reducing drug,  alcohol and tobacco use | 1 □ | 2 □ | 3 □ |
|  | Avoidance of exposure to environmental hazards or toxin such as ionizing radiation, pesticide, lead, mercury, & pets is a concern for a women with established first trimester pregnancy not for couples planning pregnancy | 1 □ | 2 □ | 3 □ |
|  | A clinician attending clients with previous caesarian section (C/S) should advise the client to delay the next pregnancy for at least 18 months before next conception | 1 □ | 2 □ | 3 □ |
|  | Infertility screening and management is not the concern of preconception care | 1 □ | 2 □ | 3 □ |

**PART THREE: Preconception Care (PCC) Practice related questions**

1. **Do you ask for the reproductive life plan (RPL) of clients attending to your day to day practice?**
   1. □ Never
   2. □ Rarely
   3. □ Sometimes
   4. □ Often times
   5. □ Always
2. **If you at least rarely ask for RPL, whom do you asking? (**select all that can apply and add if you have more)
   1. □ Adult males ( ≥ 19 years)
   2. □ Adolescents and Teens (12-18 Years)
   3. □ All adult females (≥ 19 years)
   4. □ Indicate if other___________________

**3.1. Do you in your practice, for a client contemplating/planning pregnancy, give *counseling* about issues listed from Qn # 303 - 315?**

|  | About… | **Never** | **Rarely** | **Sometimes** | **Often** | **Always** |
| --- | --- | --- | --- | --- | --- | --- |
|  | Family planning methods | 1 □ | 2 □ | 3 □ | 4 □ | 5 □ |
|  | Pregnancy spacing | 1 □ | 2 □ | 3 □ | 4 □ | 5 □ |
|  | Physical exercise | 1 □ | 2 □ | 3 □ | 4 □ | 5 □ |
|  | Body weight | 1 □ | 2 □ | 3 □ | 4 □ | 5 □ |
|  | Nutrition | 1 □ | 2 □ | 3 □ | 4 □ | 5 □ |
|  | Alcohol tobacco, and psychoactive substance use | 1 □ | 2 □ | 3 □ | 4 □ | 5 □ |
|  | Multivitamin containing Folic acid | 1 □ | 2 □ | 3 □ | 4 □ | 5 □ |
|  | Importance of maintaining good control of any preexisting  medical conditions before conception | 1 □ | 2 □ | 3 □ | 4 □ | 5 □ |
|  | Importance of screening for STIs/HIV | 1 □ | 2 □ | 3 □ | 4 □ | 5 □ |
|  | Dangers of prescribed and non prescribed medication use | 1 □ | 2 □ | 3 □ | 4 □ | 5 □ |
|  | Environmental hazard & toxins | 1 □ | 2 □ | 3 □ | 4 □ | 5 □ |
|  | Preventive vaccines | 1 □ | 2 □ | 3 □ | 4 □ | 5 □ |
|  | The importance of inviting partner for preconception  counseling, risk screening and management | 1 □ | 2 □ | 3 □ | 4 □ | 5 □ |

**3.2**. Do **you in your practice, for a client contemplating, conduct the Following preconception health assessment (Qn # 316- 328) to find preconception health risk factors?**

|  |  | **Never** | **Rarely** | **Sometimes** | **Often** | **Always** |
| --- | --- | --- | --- | --- | --- | --- |
|  | Demographic information/ | 1 □ | 2 □ | 3 □ | 4 □ | 5 □ |
|  | Past Obstetric & Gynecologic history | 1 □ | 2 □ | 3 □ | 4 □ | 5 □ |
|  | Past medical and surgical history | 1 □ | 2 □ | 3 □ | 4 □ | 5 □ |
|  | Genetic history or family pedigree | 1 □ | 2 □ | 3 □ | 4 □ | 5 □ |
|  | History of dental care/checkup | 1 □ | 2 □ | 3 □ | 4 □ | 5 □ |
|  | Social history particularly lifestyle behaviors | 1 □ | 2 □ | 3 □ | 4 □ | 5 □ |
|  | Exposure to environmental toxins and contaminants | 1 □ | 2 □ | 3 □ | 4 □ | 5 □ |
|  | Pharmacologic history | 1 □ | 2 □ | 3 □ | 4 □ | 5 □ |
|  | Nutritional assessment particularly BMI | 1 □ | 2 □ | 3 □ | 4 □ | 5 □ |
|  | Psycho-social assessment | 1 □ | 2 □ | 3 □ | 4 □ | 5 □ |
|  | Physical examination | 1 □ | 2 □ | 3 □ | 4 □ | 5 □ |
|  | Employment history | 1 □ | 2 □ | 3 □ | 4 □ | 5 □ |
|  | Vaccination status | 1 □ | 2 □ | 3 □ | 4 □ | 5 □ |

**3.3. Do you in your practice, for a client planning pregnancy, carryout the following intervention either yourself or by referring or transferring the client to other department/health facility where the client/s get services indicated in the table below?**

|  |  | **Never** | **Rarely** | **Sometimes** | **Often** | **Always** |
| --- | --- | --- | --- | --- | --- | --- |
|  | Folic acid supplementation/prescription | 1 □ | 2 □ | 3 □ | 4 □ | 5 □ |
|  | Substance use cessation. Eg. alcohol, cigarette, or other drug | 1 □ | 2 □ | 3 □ | 4 □ | 5 □ |
|  | Select safe medication or substitute the existing with safe one | 1 □ | 2 □ | 3 □ | 4 □ | 5 □ |
|  | Ordering/checking routine preconception lab investigations | 1 □ | 2 □ | 3 □ | 4 □ | 5 □ |
|  | Diagnosing & managing acute or chronic preconception risk  conditions | 1 □ | 2 □ | 3 □ | 4 □ | 5 □ |
|  | Controlling existing pre-gestational chronic diseases | 1 □ | 2 □ | 3 □ | 4 □ | 5 □ |
|  | Vaccination of client as per the national protocol | 1 □ | 2 □ | 3 □ | 4 □ | 5 □ |
|  | Pregnancy confirmation | 1 □ | 2 □ | 3 □ | 4 □ | 5 □ |
|  | Linking client to other relevant department or organizations | 1 □ | 2 □ | 3 □ | 4 □ | 5 □ |
|  | Provider initiated HIV testing and counseling (PIHTC) | 1 □ | 2 □ | 3 □ | 4 □ | 5 □ |

**PART FOUR: HP’s levels of Agreement/Disagreement on selected PCC issues:**

|  |  | **Strongly**  **Disagree** | **Disagree** | **Undecided** | **Agree** | **Strongly**  **Agree** |
| --- | --- | --- | --- | --- | --- | --- |
|  | Omission of preconception care leads to an irreversible  damage to the fetus | 1 □ | 2 □ | 3 □ | 4 □ | 5 □ |
|  | PCC provides a greatest opportunity to optimize couples health particularly women’s health before conception | 1 □ | 2 □ | 3 □ | 4 □ | 5 □ |
|  | Providing PCC service to developing countries like  Ethiopia is a luxury service | 1 □ | 2 □ | 3 □ | 4 □ | 5 □ |
|  | A hospital is not the best place to provide PCC | 1 □ | 2 □ | 3 □ | 4 □ | 5 □ |
|  | In developing country like Ethiopia, the focus of PCC should not be directed to healthy people but for people with infectious disease like HIV and HBV | 1 □ | 2 □ | 3 □ | 4 □ | 5 □ |
|  | Providing PCC is not within the scope of my professional responsibility and accountability. | 1 □ | 2 □ | 3 □ | 4 □ | 5 □ |
|  | Due to the presence of other competing demands, providing PCC is not the priority intervention I should provide. | 1 □ | 2 □ | 3 □ | 4 □ | 5 □ |
|  | Preconception care should be given for all healthy and sick individuals including those presented with critical and emergency condition. | 1 □ | 2 □ | 3 □ | 4 □ | 5 □ |
|  | All healthcare providers (professionals) can easily integrate the elements of PCC in their daily practice to all eligible individuals whom they are caring | 1 □ | 2 □ | 3 □ | 4 □ | 5 □ |
|  | Pre conception health is part of the reproductive and  human right issue to which the health professional is  responsible either for omission or commission of PCC | 1 □ | 2 □ | 3 □ | 4 □ | 5 □ |

**Instruction**: Please read each questions listed from Qn # 401- 410) and respond to each questions by mentioning your level of agreement or disagreement by ticking the box of the options indicated as 1 => *Strongly* *Disagree*, 2=> *Disagree*, 3=> *Neutral or Undecided*, 4 => *Agree*, and 5=> *Strongly disagree*.

**PART FIVE: Additional factors associated with PCC practice**

**Have you taken training on or thought about topics listed from (Select all that can apply) Qn # 501 - 509?**

|  |  | **Yes during my stay at university College**  **(Pre-service training)** | **Yes as an in-service training** | **Never ever get the training** | **Don’t remember** |
| --- | --- | --- | --- | --- | --- |
|  | Reproductive life plan screening & brief counseling | 1 □ | 2 □ | 3 □ | 4 □ |
|  | the importance of increasing public awareness Preconception health &PCC | 1 □ | 2 □ | 3 □ | 4 □ |
|  | how to conduct preconception risk assessment | 1 □ | 2 □ | 3 □ | 4 □ |
|  | how to provide preconception educational & counseling | 1 □ | 2 □ | 3 □ | 4 □ |
|  | how to manage identified preconception risk factors | 1 □ | 2 □ | 3 □ | 4 □ |
|  | the elements of interconception care needed to prevent APO | 1 □ | 2 □ | 3 □ | 4 □ |
|  | About HIV/AIDS Testing and management (E.g. PMTCT, PIHCT,VCT, or ART) | 1 □ | 2 □ | 3 □ | 4 □ |
|  | PCC considerations for clients with other chronic diseases | 1 □ | 2 □ | 3 □ | 4 □ |
|  | how to provide alcohol or tobacco cessation service | 1 □ | 2 □ | 3 □ | 4 □ |

**PART SIX: Regarding actual & potential access to resources and Hp practicing PCC**

|  |  | **Yes** | **No** |  |
| --- | --- | --- | --- | --- |
|  | Do you have access to internet | □ 1 | □ 2 | If "No" go to Qn# 603 |
|  | From do you get internet access?  □ 1. Office or library  **( Select all that can apply )** □ 2 From internet cafe  □ 3 From hotel WIFI service  □ 4 Mobile phone /CDMA/ internet |  |  |  |
|  | Do you use your smart phone(SP) to share e-resources from others | □ 1 | □ 2 | □ 3 I Don’t have SP |
|  | Does your institution have library? | □ 1 | □ 2 |  |
|  | Does your institution have policy and procedural document guiding PCC? | □ 1 | □ 2 | □ 3 I Don’t know |
|  | Did you, so far, get or see any PCC gridline or protocol from any source? | □ 1 | □ 2 |  |
|  | Have you ever seen national PCC guideline or protocol prepared by FMOH? | □ 1 | □ 2 | □ 3 I Don’t know |
|  | Have you seen any HP practicing PCC in your facility? | □ 1 | □ 2 |  |
|  | Are you willing to incorporate elements of PCC in your daily practice? | □ 1 | □ 2 | □ 3 Undecided |
|  | Do you want training on PCC? | □ 1 | □ 2 |  |

1. **Whom do you recommend to provide preconception care (Select all that can apply & add if more)**
   1. □ All Specialist Doctors
   2. □ All General Practitioners
   3. □ All Nurses
   4. □ All Midwifes
   5. □ Health Officers
   6. □ Health Extension workers
   7. Others._________________
2. In which facility should preconception care service be given (select all that can apply and add if you suggest other)
   1. □ Health center
   2. □ Hospital
   3. □ Other. _____________________________
3. If you provide PCC in the past three months, for how many times you provide PCC? ____ times in the past three months (please write it in number)

Thank you for Participating in this study!!!

| **General Information (To be filled by Research Assistant and Supervisors)** | | |
| --- | --- | --- |
| Health Facility Name |  | Are all pages checked for availability and completeness? |
| Date the questionnaire was completed | /_________/ 2017 | Remark by Datatec collector |
| Data collector name  Signature | ____________ |  |
| HP’s Department | ______________________________ |  |
| Codes/PHI – UCSC(Eg. 00/00/ - 000) | __________/___________/ - ______________ | Remark by Supervisor |
| Name of the Supervisor  Signature | ____________ |  |
| Date checked by supervisor | _____/_______/ 2017 |  |
